# Editorial: Osteoporosis and adipokines: the potential for future treatment

**DOI:** 10.3389/fendo.2024.1405412

**Published:** 2024-04-10

**Authors:** Saba Tariq, Sundus Tariq, Mukhtiar Baig, Amina Valjevac

**Affiliations:** ^1^ Institute of Metabolism and Systems Research, University of Birmingham, Birmingham, United Kingdom; ^2^ Department of Pharmacology, University Medical and Dental College, The University of Faisalabad, Faisalabad, Pakistan; ^3^ Department of Physiology, International School of Medicine, Istanbul Medipol University, Research Institute for Health Sciences and Technologies (SABITA), Istanbul, Türkiye; ^4^ Department of Clinical Biochemistry, Faculty of Medicine, King Abdulaziz University, Jeddah, Saudi Arabia; ^5^ Department of Human Physiology, Faculty of Medicine, University of Sarajevo, Sarajevo, Bosnia and Herzegovina

**Keywords:** osteoporosis, adipokines, BMD, treatment, women

Osteoporosis is a major health concern, particularly in older people. It is characterized by decreased bone density and a higher fracture risk. Globally, it affects 35.3% of women and 12.5% of men (1). Recent studies have concentrated on the role of adipokines in bone metabolism control and osteoporosis progression. Adipokines are signaling substances produced by adipose tissue. Leptin, resistin, chemerin, visfatin, and other proteins have been shown to alter bone metabolism, making them possible targets for future osteoporosis treatments (2–4).

Understanding the link between adipokines and bone health may lead to specific medicines that strengthen bones and reduce fracture risk in osteoporosis patients. However, the current literature yields inconsistent outcomes. More research and clinical trials are required to thoroughly understand the therapeutic potential of adipokines in controlling this common skeletal condition. However, because adipokines are linked to bone remodeling, inflammation, and hormonal control in a complex way, targeting them may be a good way to treat osteoporosis in the future. The present study topic has five accepted publications, with the key points mentioned below:

Wang et al. looked at the relationship between blood adipokine levels (leptin, adiponectin, and chemerin) and the prevalence of senile osteoporosis (SOP) in their meta-analysis. After reviewing eleven pertinent studies, they concluded that, in comparison to older, healthy people with appropriate bone density, SOP patients had lower serum leptin levels and higher levels of chemerin, while their levels of adiponectin were constant. Bone density and leptin levels had a positive association; however, chemerin levels indicated a negative correlation. Furthermore, leptin and adiponectin levels were strongly correlated with body mass index (BMI). These results implied that lower adiponectin and leptin levels were linked to lower BMI and that those with higher chemerin and lower leptin levels were more likely to have SOP.

Using NHANES data from 2007 to 2020, Sun et al. conducted a cross-sectional study to examine the relationship between osteoporosis and femur bone mineral density (BMD) in older Americans. The study discovered that while an increase in Visceral Adipose Index (VAI) values was linked to a decreased incidence of osteoporosis, greater VAI values were associated with a higher incidence. Positive correlations between femur BMD and VAI were also seen, displaying intricate patterns that may indicate limits beyond which excessive visceral fat accumulation could have detrimental effects on bone health.

A study by Kang et al. looked into possible mediating human factors to provide light on how lipid metabolism affects bone metabolism. Genetic data from large-scale research on blood lipids and bone mineral density (BMD) were analyzed using Mendelian randomization. According to the results, higher levels of TC, LDL-C, and HDL-C were linked to lower lumbar spine BMD. Evidence shows that hand grip strength (HGS) mediates the relationship between systolic blood pressure (SBP) and HGS. Overall, it was determined that there is a negative causal link between lipid and bone metabolism, with SBP and HGS acting as mediators in this interaction.

Using Mendelian randomization (MR) analyses, Zhou et al. sought to determine the causal relationship between osteoporosis (OP) and non-alcoholic fatty liver disease (NAFLD). Findings pointed to a link between OP and genetically predicted NAFLD, which includes imaging-based liver fat content and biopsy-confirmed NAFLD. A similar tendency was observed in chronically high serum alanine aminotransferase, although this was not statistically significant. Sensitivity analysis confirmed that the results were reliable. Overall, the data suggests that OP and NAFLD may be causally related.

Finally, a mini review by Patil et al. comprehends how new adipokines affect the prognosis of osteoporosis (OP) in order to develop effective treatment plans. Current research demonstrates associations between particular adipokines and bone loss, demonstrating a dynamic link between muscle and bone at the biochemical level. Understanding the molecular messages that adipose tissue and bone exchange highlight the pathophysiological commonalities among age-related diseases. It was advised to focus on pre-clinical studies involving human subjects to assess the therapeutic potential of adipokines in the management of OP. Adipokines are also crucial for understanding and even treating comorbidities like obesity, diabetes, and inflammation. They may offer new ways to treat these illnesses along with OP.

Osteoporosis is a complicated and expensive condition to treat; it is necessary to discover modifiable risk factors in order to create future treatments. The purpose of this Research Topic was to clarify the vital role adipokines play in osteoporosis prevention and development. Through examining the effects of adipokines on bone strength, density, and structure, the Research Topic aimed to identify the underlying causes of osteoporosis. Additionally, researching adipokines as possible therapeutic targets aims to create novel therapy approaches for better controlling or preventing osteoporosis (5, 6). The goal of these initiatives was to enhance the results of osteoporosis treatment by focusing on particular adipokine-influenced pathways, which may lessen the severity of this disease.

## Author contributions

SaT: Conceptualization, Writing – original draft, Writing – review & editing. SuT: Writing – review & editing. MB: Writing – review & editing. AV: Writing – review & editing.
